# Human-Induced Pluripotent Stem Cell Models for Amyloid Cardiomyopathy: From Mechanistic Insights to Therapeutic Discovery

**DOI:** 10.3390/jcdd12110434

**Published:** 2025-11-02

**Authors:** Yufeng Liu, Muhammad Riaz

**Affiliations:** 1 Department of Internal Medicine, Yale Cardiovascular Research Center, Section of Cardiovascular Medicine, Yale University School of Medicine, New Haven, CT 06511, USA; yufeng.liu@yale.edu; 2 Vascular Biology and Therapeutics Program, Yale University School of Medicine, New Haven, CT 06520, USA; 3 Department of Pathology, Yale University School of Medicine, New Haven, CT 06520, USA

**Keywords:** amyloid cardiomyopathy, transthyretin amyloidosis, light chain amyloidosis, induced pluripotent stem cell-derived cardiomyocytes, induced pluripotent stem cell-derived hepatocytes, 3D tissue engineering, genome editing, drug screening

## Abstract

Amyloid cardiomyopathy (ACM), driven by transthyretin (TTR) and immunoglobulin light chain (LC) amyloid fibrils, remains a major clinical challenge due to limited mechanistic understanding and insufficient preclinical models. Human-induced pluripotent stem cells (iPSCs) have emerged as a transformative platform to model ACM, offering patient-specific and genetically controlled systems. In this review, we summarize recent advances in the use of iPSC-derived cardiomyocytes (iPSC-CMs) in both two-dimensional (2D) monolayer cultures and three-dimensional (3D) constructs—including spheroids, organoids, cardiac microtissues, and engineered heart tissues (EHTs)—for disease modeling, mechanistic research, and drug discovery. While 2D culture of iPSC-CMs reproduces hallmark proteotoxic phenotypes such as sarcomeric disorganization, oxidative stress, and apoptosis in ACM, 3D models provide enhanced physiological relevance through incorporating multicellularity, extracellular matrix interactions, and mechanical load-related features. Genome editing with Clustered Regularly Interspaced Short Palindromic Repeats (CRISPR)-Cas9 further broadens the scope of iPSC-based models, enabling isogenic comparisons and the dissection of mutation-specific effects, particularly in transthyretin-related amyloidosis (ATTR). Despite limitations such as cellular immaturity and challenges in recapitulating aging-associated phenotypes, ongoing refinements in differentiation, maturation, and dynamic training of iPSC-cardiac models hold great promise for overcoming these barriers. Together, these advances position iPSC-based systems as powerful human-relevant platforms for modeling and elucidating disease mechanisms and accelerating therapeutic development to prevent ACM.

## 1. Introduction

Amyloid cardiomyopathy (ACM) is a progressive, often underrecognized condition characterized by the extracellular deposition of insoluble misfolded proteins, which form aggregates known as amyloid fibrils in the heart, leading to restrictive cardiomyopathy, arrhythmias, and heart failure [1,2,3,4]. This situation is worsened in older adults, with substantial diagnostic delays and poor prognosis if untreated [5].

Historically, ACM dates back to the early 20th century, when the term “amyloidosis” was first coined to describe the accumulation of amyloid proteins in various tissues, predominantly as neuropathic phenotypes [6]. Early recognition of cardiac involvement was challenging due to its subtle and nonspecific symptoms, often mistaken for other forms of heart disease [7]. In 1922, Congo red, an aniline dye, was introduced as a diagnostic tool for identifying amyloid deposits, marking a significant advancement in the field of amyloid research. This dye selectively binds to amyloid fibrils, and when used in tissue staining, it improves the reliability of diagnosing cardiac amyloid deposits. Despite this, it wasn’t until the 1950s that electron microscopy first revealed amyloid fibrils, showing a distinct structure different from collagen and consistent across tissues, advancing the understanding of amyloid’s ultrastructure [8]. In the 1960s, Pras and colleagues developed a water extraction method that isolated amyloid fibrils and identified their β-pleated sheet configuration, thereby enhancing the understanding of their biochemical structure [9].

While cardiac amyloidosis was once considered rare due to its subtle symptoms, its clinical significance has grown with technological advancements. Specifically, imaging techniques such as echocardiography, cardiac magnetic resonance imaging (cMRI), and nuclear imaging have allowed for earlier and more accurate detection of cardiac involvement, including increased left ventricular wall thickness, myocardial speckling, and late gadolinium enhancement, which are indicative of amyloid deposition [2,10,11]. In addition, biopsy techniques, including endomyocardial and extracardiac biopsies, along with the use of biomarkers such as NT-proBNP and cardiac troponin, help in confirming the diagnosis and assessing the severity of the disease.

Traditional transplantation methods provide a choice of therapy at an early stage with high cost [2,12]; however, more specific therapies depending on the underlying type of amyloidosis are now available [13,14]. While cardiac management involves standard heart failure therapies, these are often poorly tolerated in ACM due to hypotension and bradycardia. Early detection and targeted treatment are crucial for improving outcomes, as heart failure and arrhythmias are significant causes of morbidity and mortality in affected individuals [15].

## 2. Amyloid Cardiomyopathy Types

**Figure 1:**
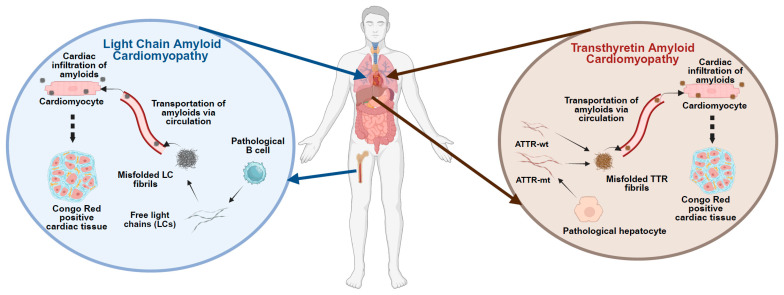
**Pathological features of Amyloid Cardiomyopathy.** The figure illustrates the distinct molecular and cellular pathways underlying the development of amyloid cardiomyopathy. Left panel: In light chain (AL) amyloidosis, monoclonal plasma cells in the bone marrow produce misfolded immunoglobulin light chains, which circulate and infiltrate as amyloid fibrils in the myocardium, leading to structural and functional cardiac impairment. Right panel: In transthyretin (ATTR) amyloidosis, either wild-type or mutant transthyretin (TTR) produced by the liver undergoes destabilization and misfolding, resulting in amyloid fibril deposition in cardiac tissue. Both pathways ultimately result in restrictive cardiomyopathy and progressive heart failure. Source organs and cell types, along with target cardiac tissues, are depicted.

Over the years, the classification of amyloidosis expanded to include various types, with light-chain amyloidosis (AL) and transthyretin-related amyloidosis (ATTR) being the most prominent forms associated with cardiac involvement, as shown in Figure 1. ATTR was further divided into wild-type (ATTR-wt) and mutant (ATTR-mt) forms, with the latter being genetically inherited [3]. By providing an overview of AL and ATTR-wt as background, this review primarily focuses on the ATTR-mt and emphasizes the potential of patient-derived induced pluripotent stem cells (iPSCs) as a versatile platform for disease modeling. Through directed differentiation of iPSCs into relevant target cell types, particularly cardiomyocytes and hepatocytes, under both two-dimensional (2D) and three-dimensional (3D) culture conditions, these models enable the recapitulation of disease phenotypes, the investigation of underlying molecular mechanisms, and the facilitation of preclinical drug screening, thereby advancing the development of targeted therapeutic strategies for ATTR amyloidosis.

### 2.1. AL Cardiomyopathy

AL is a result of the deposition of amyloid fibrils composed of immunoglobulin light chais (LCs) in the heart. These LCs are typically produced by a monoclonal plasma cell disorder, such as multiple myeloma or Waldenström’s macroglobulinemia [16] Figure 1. AL-ACM involves both direct proteotoxic effects of circulating free LCs and the mechanical and metabolic consequences of amyloid infiltration into the myocardium. This dual mechanism leads to progressive cardiomyocyte dysfunction, increased wall stiffness, diastolic heart failure, arrhythmias, and poor prognosis, particularly when cardiac involvement is detected late [13]. Toxic light chains undergo misfolding and fibril formation, impairing intracellular homeostasis by disrupting mitochondrial function and proteostasis, which leads to energy deficits and accumulation of misfolded proteins. They also induce endoplasmic reticulum stress, oxidative damage, and autophagic dysfunction, further contributing to cardiomyocyte injury and cell death [17,18]. These intracellular toxicities act synergistically with extracellular amyloid burden to accelerate cardiac decline in AL-ACM [19,20].

On the diagnostic front, AL-ACM diagnosis is commonly based on the imaging evidence, confirmed through Congo red staining of endomyocardial or extracardiac biopsies, supported with serum/urine free light chain measurements, immunofixation, and cardiac biomarkers (e.g., NT-proBNP, troponins). Importantly, the degree of cardiac involvement is the most critical determinant of prognosis, and even modest myocardial infiltration by amyloid can lead to fatal outcomes if it remains untreated [21].

Therapeutic strategies focus on reducing the source of amyloidogenic LCs through anti–plasma cell therapies. Chemotherapy regimens, often using agents like melphalan and dexamethasone, aim to suppress clonal plasma cells’ activity [22]. More targeted approaches, such as bortezomib-based therapies and monoclonal antibodies like daratumumab, are designed to achieve a hematologic complete response (CR), which is strongly associated with improved survival outcomes. Bortezomib acts as a proteasome inhibitor that rapidly reduces LC levels [13,23,24], while daratumumab targets CD38 on plasma cells [25], offering more profound and sustained responses, especially in patients with refractory or high-risk disease. In some cases, autologous stem cell transplantation (ASCT) may be considered for eligible patients with less advanced cardiac involvement, with the goal of achieving a hematological response—a key predictor of survival [26]. In addition to chemotherapy, heart failure management is crucial, although it is often complicated by the unique hemodynamics of ACM [21]. Advanced therapies and supportive care can improve the prognosis of AL ACM, but the disease remains still challenging to manage because of its systemic nature and the severity of cardiac involvement at diagnosis. Given the limited number of iPSC-based models for AL amyloidosis available, the current review primarily emphasizes ATTR, particularly hereditary ATTR-mt, while briefly summarizing the key mechanisms of AL-ACM. Readers are referred to comprehensive reviews by Lavatelli et al. [18] for in-depth discussion.

### 2.2. ATTR Cardiomyopathy

ATTR cardiomyopathy is a form of amyloidosis where the transthyretin (TTR) protein forms misfolded fibrils that deposit in the heart. TTR is a 127-amino-acid tetrameric protein, primarily produced in the liver and secreted into the bloodstream. It binds to thyroid hormone thyroxine (T4) and retinol (vitamin A) via retinol binding protein and transports this complex to all organs throughout the body [27]. In a disease scenario, it can, however, undergo aggregation into amyloid fibrils that infiltrate into the myocardium, contributing to myocardial stiffness, arrhythmias, and heart failure Figure 1. ATTR amyloidosis is divided into two subtypes: 1. ATTR-wt amyloidosis, caused by aging-related misfolding of the wild-type TTR protein. 2. ATTR-mt amyloidosis occurs due to misfolding of the mutant TTR protein caused by hereditary mutations in the TTR gene [28] Figure 1.

#### 2.2.1. ATTR-wt

ATTR-wt is a systemic condition characterized by amyloid deposits in the heart, ligaments, tendons, lungs, and intestines, predominantly affecting individuals over 65 years old [29]. Additionally, recent clinical data indicate that among patients over 75 with confirmed ATTR-wt, 75% were classified as frail based on the G8 screening tool [30] and another study based on autopsy data demonstrated that TTR amyloidosis occurs in the left ventricular myocardium in 32% of patients who are older than 75 years old with heart failure with preserved ejection fraction (HFpEF) [31], highlighting the intersection between aging, frailty, and disease burden in this population. Mechanistically, ATTR-wt leads to the deposition of non-mutated TTR protein in the myocardial extracellular space, leading to restrictive cardiomyopathy, arrhythmias, and heart failure. Biochemically, cardiac amyloid deposits often contain both full-length TTR and C-terminal TTR fragments, with cleavage at positions like 48, 49, or 81 enhancing amyloidogenicity under physiological shear stress [32]. Factors such as oxidative modifications (especially at Cys-10) [33,34], impaired chaperone activity (e.g., clusterin) [35], and protease-mediated cleavage [36] are believed to contribute to the destabilization of TTR wild-type tetramers and fibril formation in the heart. Histologically, ATTR-wt fibrils are short and disorganized (type A), contrasting with the long, aligned fibrils (type B) seen in early-onset ATTR-mt amyloidosis [32].

ATTR-wt is typically diagnosed at an advanced stage, and treatment remains supportive, as no specific therapies are currently approved to target the amyloid deposits. Standard heart failure treatments, such as diuretics, angiotensin-converting enzyme (ACE) inhibitors, and beta-blockers, are used; however, these therapies often have limited efficacy due to the unique pathophysiology of ATTR-wt, which includes reduced cardiac output and impaired myocardial relaxation [12]. Therapeutically, stabilizers like tafamidis have demonstrated survival benefits [37]. In addition, gene-silencing strategies targeting hepatic TTR production (e.g., siRNA and antisense oligonucleotides) have also been approved for clinical application; however, limited patient-associated efficacy data [38].

#### 2.2.2. ATTR-mt

ATTR-mt is associated with autosomal dominant inherited mutations in the TTR gene that result in the production of misfolded TTR proteins, which aggregate into amyloid fibrils and deposit in the heart. This type of ACM is commonly seen in individuals with genetic mutations such as the most common Val30Met mutation [39]. Treatment for ATTR-mt has significantly advanced with the approval of tafamidis, a drug that stabilizes the TTR tetramer and prevents its misfolding and subsequent amyloid fibril formation, thereby improving survival and reducing the progression of heart failure, particularly in individuals with the Val30Met mutation. In addition, supportive treatments for heart failure, including diuretics, ACE inhibitors, and beta-blockers, are employed, although caution is needed due to the potential for hypotension and bradycardia [2,12,28,40]. The combination of disease-modifying therapy and heart failure management provides a promising management for patients with ATTR-mt, especially if the disease is diagnosed early [41].

## 3. Generation of Patient-Specific iPSCs and Target Derivatives as a Model System for ACM

**Figure 2:**
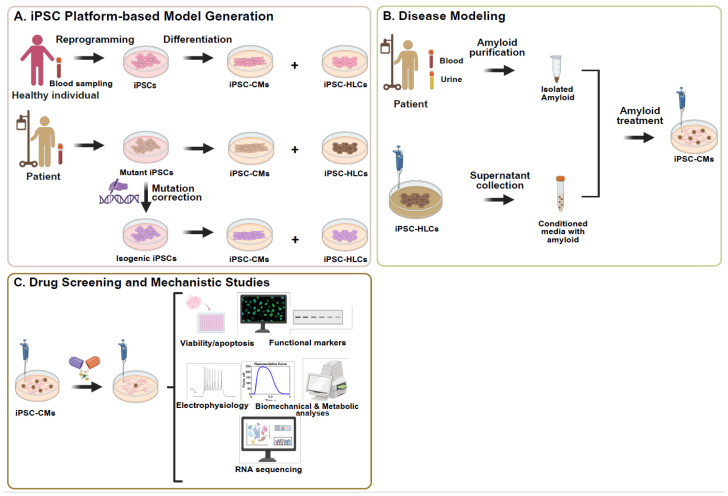
**iPSC-based platform for modelling TTR-related amyloid cardiomyopathy, drug screening, and mechanistic studies.** (**A**) iPSCs are generated from peripheral blood mononuclear cells obtained from a blood sample drawn from either a healthy individual or a patient with ATTR. (**B**) Amyloid cardiomyopathy phenotypes are modeled in vitro by treating iPSC-derived cardiomyocytes (iPSC-CMs) with either (i) patient-derived amyloid fibrils isolated from serum or urine, or (ii) conditioned medium from the patient-specific iPSC-HLCs secreting pathogenic mutant TTR protein. (**C**) Treated iPSC-CMs are used for high-throughput drug screening and mechanistic studies to identify disease-related pathways and evaluate candidate therapeutic compounds. This platform provides a human-relevant, patient-specific model for studying the progression of amyloid cardiomyopathy and therapeutic response. Abbreviations: ACM, amyloid cardiomyopathy; TTR, transthyretin; iPSC-HLCs, iPSC-derived hepatocyte-like cells; iPSC-CMs, iPSC-derived cardiomyocytes.

Recognizing the absence of suitable animal models that replicate the multisystem complexity and clinical variability of this condition [42], the advent of patient-specific iPSCs has transformed the landscape of cardiac disease modeling, offering an unprecedented opportunity to recapitulate the genetic and molecular underpinnings of ACM in vitro. It is a scalable and ethically acceptable platform, as they are easy to expand, readily available, and do not require the destruction of embryos, thereby mitigating common concerns associated with embryonic stem cell use. This makes them especially valuable for studying TTR amyloidosis, where hepatocytes are the primary source of TTR secretion and cardiomyocytes are the major target of fibril deposition Figure 2A.

### 3.1. iPSCs Offer Genetic Context for Studying the Role of TTR Gene Mutations in Amyloidosis

iPSCs provide a uniquely powerful system for modeling hereditary ATTR by enabling the derivation of multiple disease-relevant cell types, including cardiomyocytes and hepatocytes, from a single individual while preserving their complete genetic and epigenetic identity. Early methods for generating iPSCs relied on integrating viral vectors, such as retroviral or lentiviral systems, to deliver the core reprogramming transcription factors OCT4, SOX2, KLF4, and c-MYC into somatic cells—most commonly skin fibroblasts [43]. Although these approaches were relatively efficient, they posed a risk of insertional mutagenesis due to random genomic integration. To overcome these limitations, non-integrating strategies were subsequently developed, including episomal plasmids, Sendai virus vectors, and mRNA or protein delivery systems. Among these, the Sendai virus vector—an RNA virus that replicates exclusively in the cytoplasm and is naturally eliminated after several passages—has become the most widely adopted and standardized method for generating both research-grade and clinical-grade iPSCs due to its high efficiency and safety profile [44]. More recent approaches employ small molecules [45] or recombinant proteins [46] to induce pluripotency without genetic modification, though they often require longer reprogramming periods and yield lower efficiencies. Regardless of the method, these advances have enabled the derivation of iPSCs from diverse patient cell sources, including peripheral blood mononuclear cells [47] and urine-derived epithelial cells [48,49], thereby establishing a scalable and ethically acceptable platform for producing patient-specific iPSCs that can be expanded indefinitely and differentiated into disease-relevant lineages such as cardiomyocytes and hepatocytes [50] Figure 2A. For detailed procedural standards, recent consensus protocols and guidelines on human iPSC generation and characterization provide comprehensive methodological recommendations [51].

This patient-specific approach is particularly valuable for studying hereditary ATTR, where over 150 mutations in the TTR gene—located on chromosome 18 and comprising four exons—have been identified [52,53,54]. Most of these mutations are pathogenic point mutations, with only a few being non-amyloidogenic [39]. Notably, some variants, such as Thr119Met, may even be protective; for example, compound heterozygosity with both Val30Met and Thr119Met has been associated with delayed disease onset by stabilizing TTR tetramers [55]. The Val30Met and Val122Ile mutations remain the most frequently observed and well-characterized globally. However, the spectrum of TTR mutations is highly heterogeneous and varies significantly by geographic and ethnic background. For example, Val30Met is endemic in regions such as Portugal, Sweden, Japan, Majorca, and Cyprus [39], and Val122Ile is predominantly found in individuals of African descent [56], while Thr60Ala is found in Northern Ireland and Irish populations, Glu89Gln in Bulgaria, Ser50Arg in Mexico, Phe64Leu in Sicily, Ser77Tyr/Phe in France, and Ala97Ser in Taiwan [39]. Many mutations have also been documented in isolated families, often with incomplete penetrance that increases with age and can vary based on factors such as genetic background, sex of the transmitting parent, and region of origin [57].

Notably, many mutations in the TTR gene lead to multisystemic involvement and influence the tropism of specific organs. Distinct amino acid substitutions alter protein stability and tissue deposition, producing variable phenotypes ranging from cardiac amyloidosis (e.g., Val122Ile) to neuropathic forms (e.g., Val30Met) [58,59]. However, this tropism is not absolute, as many variants show mixed organ involvement [60]. The molecular mechanisms likely involve differences in tetramer dissociation, proteolytic processing, and tissue-specific chaperone or clearance pathways [61]. Additionally, modifier genes, epigenetic, and environmental factors influence disease heterogeneity and progression [62]. Developing integrated, modular models to capture these genetic and molecular interactions may improve understanding of tropism and enable precision diagnosis and personalized therapy in ATTR amyloidosis [63].

The iPSC-based disease modeling offers a powerful platform to study this genetic and phenotypic heterogeneity Figure 2B. By reprogramming somatic cells from patients carrying different TTR mutations—across diverse countries, ancestries, and clinical phenotypes—a comprehensive iPSC biobank capturing the patient’s unique genetic background can be constructed [64]. For instance, several groups have successfully established iPSC lines from ATTR patients carrying pathogenic heterozygous mutations in the TTR gene—such as Val30Met [65], Phe53Val [66], Asp38Asn [67], and Ser43Asn [68]. These patient-derived iPSCs exhibit hallmark characteristics of pluripotency, including the expression of stemness markers (e.g., OCT4, NANOG, TRA-1-60), normal karyotype, and in vivo teratoma formation with contributions to all three germ layers. Building on this foundation, Giadone et al. developed a large, ethnically diverse (including African American, Caucasian, and Asian populations) iPSC library from over 30 ATTR patients carrying TTR mutations such as Val30Met, Val122Ile, Leu55Pro, and Iso107Met, representing a range of clinical phenotypes including neuropathy and cardiomyopathy [69]. Similarly, Leung et al. generated patient-specific iPSC lines from an individual with the aggressive Leu55Pro mutation to model mutation-specific cellular toxicity. In both cases, the iPSCs served as a renewable and genetically faithful platform for investigating disease mechanisms in a patient-matched context [70] Figure 2B.

Together, these efforts would allow researchers to systematically investigate genotype–phenotype relationships, molecular mechanisms of mutation-specific amyloidogenesis, and the influence of genetic modifiers in a controlled and human-relevant system. Importantly, such a resource would also enable side-by-side comparisons of mutant versus wild-type TTR expression, secretion, and aggregation behaviors in hepatocytes (the primary source of circulating TTR) and cardiomyocytes (key targets of amyloid toxicity). As these libraries expand, this approach lays the groundwork for personalized medicine by facilitating the screening of mutation-specific therapies, optimizing patient stratification for clinical trials, and identifying novel therapeutic targets tailored to different genotypes and patient populations. Based on the above iPSC sources, they can be further differentiated into both hepatocyte-like cells (HLCs) and cardiomyocytes for systematic disease modeling, which will be introduced in Section 3.2, Section 3.3, Section 3.4 and depicted in Figure 2.

### 3.2. Derivation of Functional Hepatocytes from iPSCs

The pathogenesis of ACM critically involves the liver, as it is the primary site of TTR synthesis. To recapitulate this hepatic-cardiac axis in vitro, iPSCs can be robustly directed into hepatic lineages, including HLCs, which secrete both wild-type and mutant forms of TTR Figure 2A,B. The differentiation protocol guiding iPSCs into hepatocytes is based on principles of embryonic liver development, in which the posterior foregut—formed from definitive endoderm—gives rise to hepatic progenitors under the influence of mesodermal signaling factors such as fibroblast growth factor 2 (FGF2) and bone morphogenetic protein 4 (BMP4) [71]. This process is recapitulated in vitro through a three-stage protocol: (1) iPSCs are first exposed to high concentrations of Activin A, often supplemented with CHIR99021 or Wnt3a, which activates Nodal and Wnt signaling pathways, respectively. This step directs the pluripotent cells toward a definitive endoderm fate, as evidenced by upregulation of key transcription factors such as SOX17, FOXA2, and CXCR4—mirroring early gut tube formation in vivo; (2) Once definitive endoderm is established, the cells are treated with FGF2 and BMP4. These signals emulate the hepatic-inductive cues secreted by the cardiogenic mesoderm in embryonic development, driving the expression of hepatic progenitor markers like alpha-fetoprotein (AFP) and hepatocyte nuclear factor 4 alpha (HNF4α); and (3) The final maturation step utilizes hepatocyte growth factor (HGF), oncostatin M, and dexamethasone, recapitulating the in vivo niche provided by the fetal liver microenvironment. This induces the expression of mature hepatic markers, including albumin (ALB), alpha-1 antitrypsin (A1AT), cytochrome P450 3A4 (CYP3A4), as well as the TTR protein, enabling the cells to perform key hepatic functions [71,72,73].

Alternative strategies, including small-molecule-only protocols, also enable rapid and cost-effective hepatic differentiation. These involve molecules that sequentially inhibit pathways such as glycogen synthase kinase 3 beta (GSK3β) and transforming growth factor beta (TGF-β)/SMAD, guiding the transition through each developmental phase with defined timing and minimal variability [74]. Some protocols also leverage partial reprogramming into hepatoblasts [75] or hepatic progenitor-like cells [72], which can expand and then be induced into mature HLCs with high efficiency and functionality. All these iPSC-derived HLCs exhibit functional characteristics, including albumin secretion, urea production, glycogen storage, cytochrome P450 activity, and indocyanine green uptake/release [71,72,73,74,75]. Notably, Asplund et al. established a standardized differentiation procedure that reliably produces homogeneous hepatocyte cultures across a large panel of hPSC lines without line-specific modifications. These hepatocytes display reproducible inter-individual variation in metabolic functions such as cytochrome P450 activity, closely reflecting primary hepatocytes from different donors. This robustness supports the generation of diverse human induced pluripotent stem cell (hiPSC)-derived hepatocyte panels for modeling metabolism, toxicity, and personalized therapies [76].

In the context of ATTR amyloidosis, iPSC-derived HLCs (iPSC-HLCs) provide a reliable platform for modeling the hepatic production of both wild-type and mutant TTR, capturing patient-specific differences in TTR expression, secretion, and downstream impact (Figure 2A,B). Studies have shown that iPSC-HLCs generated from patients carrying amyloidogenic TTR mutations—such as Leu55Pro, Val122Ile, and Val30Met—secrete both wild-type and mutant TTR into the culture medium, faithfully mimicking the heterozygous TTR production observed in vivo [69,70,77]. Notably, those mutant HLCs upregulate genes related to protein folding and stress response [70]. Those secreted TTR proteins also exhibit mutation-dependent biochemical behaviors, including differential stability, aggregation potential, and oligomer formation. For example, mutant HLCs (especially Leu55Pro) exhibit enhanced secretion of misfolded or aggregation-prone TTR species compared to wild-type HLCs. Additionally, quantitative proteomic analysis of the secretome from these iPSC-HLCs has revealed distinct expression patterns associated with specific TTR variants, providing a scalable and genotype-specific readout for assessing the molecular basis of amyloidogenicity [69]. Importantly, iPSC-HLCs also enable longitudinal assessment of TTR production over time, under defined conditions, without the need for invasive liver biopsy or limited-access primary hepatocytes. These features make HLCs an essential component in recreating the hepatic-cardiac axis of ATTR amyloidosis in vitro and studying the mutation-specific secretion and extracellular fate of TTR that underlie cardiac amyloid deposition.

### 3.3. Derivation of Functional Cardiomyocytes from iPSCs

Recent advances in differentiation protocols and culture platforms have enabled the generation of iPSC-derived cardiomyocytes (iPSC-CMs) that exhibit hallmark features of cardiac function, such as spontaneous contractility, organized sarcomeres, and functional electrophysiological activity, albeit with limitations in maturity [78,79]. Initial protocols based on embryoid body (EB) formation—originally developed in embryonic stem cells—produced beating iPSC-CMs but were limited by low purity (≤1%) due to diffusion barriers and heterogeneous signaling environments [80]. Monolayer-based differentiation methods have since become the preferred approach, offering higher reproducibility and cardiomyocyte purity. These protocols typically involve serial application of growth factors such as Activin A, BMP4, and bFGF on Matrigel-coated substrates, or employ chemically defined media (e.g., CDM3) in conjunction with Wnt/β-catenin modulation to recapitulate embryonic heart development [81]. The temporal control of these pathways enables a stage-wise transition from mesoderm induction (Wnt signaling activation) to cardiac progenitor specification (Wnt signaling inhibition) and maturation, with differentiation efficiency reaching up to 95% cardiac troponin T type 2 (TNNT2) positive cells in optimized systems [78,79]. The resulting iPSC-CMs express canonical cardiac transcription factors (e.g., NKX2.5, GATA4, TBX5) and structural proteins (TNNT2, α-actinin, and MYH6/7), and display action potentials, calcium transients, and force generation consistent with atrial-, ventricular-, or nodal-like subtypes. However, current differentiation protocols often yield a mixed cardiac population, and the presence of non-cardiac cells or subtype heterogeneity may influence downstream applications. Efforts to overcome this include manipulating retinoic acid and Wnt signaling to steer subtype specification and leveraging epigenetic memory from the tissue of origin to enrich for ventricular progenitors. Ultimately, the purity of iPSC-CMs can be enhanced through metabolic selection using lactate or by employing surface marker-based sorting techniques [82].

The platforms above that utilize HLCs and cardiomyocytes derived from the same individual can be effectively used to model ACM, as the differentiated iPSC-HLCs secrete a substantial amount of TTR protein, and iPSC-CMs retain patient-specific genotypes, exhibiting hallmark cardiac phenotypes. When exposed to the amyloidogenic TTR mutant protein either isolated from patients’ serum/urine or present in conditioned medium of mutant iPSC-HLCs, iPSC-CMs can be utilized to investigate stress responses observed in diseased myocardium, including the activation of proteostasis networks and cytotoxicity (Figure 2A,B). This enables mechanistic interrogation of mutation-specific pathogenicity and supports screening of candidate therapeutics aimed at mitigating cardiotoxic effects (Figure 2C). The use of isogenic corrected lines further strengthens their utility for dissecting the molecular consequences of specific TTR mutations in a cardiac context (Figure 2A–C).

### 3.4. Integrating iPSC-HLCs and CMs for Systematic Modeling of ATTR

By exposing iPSC-CMs to conditioned media from mutant-TTR-producing HLCs, researchers can model TTR-related toxicity and study patient-specific pathogenic mechanisms, including cardiotoxicity [69,70]. For instance, in the Leu55Pro ATTR iPSC model, HLCs were shown to produce and secrete pathogenic TTR into the culture medium, which then induced cytotoxic effects in iPSC-CMs with hallmark disease phenotypes such as oxidative stress and increased cell death upon exposure to mutant TTR. Similarly, direct application of recombinant Val122Ile TTR fibrils to iPSC-CMs led to sarcomeric disarray, loss of cell–cell coupling, and irregular beating, validating the cytotoxic potential of TTR fibrils in vitro [83]. This ability to recapitulate the entire disease cascade in vitro, from hepatic production of destabilized TTR to its cytopathic effect on cardiac or neuronal targets, represents a significant advancement over traditional models that isolate hepatic or cardiac aspects in unrelated systems. Moreover, the use of iPSCs derived from ATTR patients provides an invaluable tool for pharmacogenomic screening, as demonstrated by the efficacy of TTR-stabilizing small molecule flufenamic acid in reversing the toxic phenotypes in patient-derived cardiomyocytes exposed to mutant TTR [70]. In conclusion, these studies highlight the versatility of patient-specific iPSC platforms, enabling researchers to generate both effector and target cell types—namely, hepatocytes and cardiomyocytes—from the same individual, thereby accurately modeling the intra-individual dynamics of amyloidogenesis.

### 3.5. Patient-Specific iPSCs Enable Efficient Genetic Manipulation

Genomic manipulation provides a powerful opportunity to understand how iPSCs derived from patients with known amyloid mutations, especially TTR-ACM, can help study amyloid disease phenotypes in an isogenic background [84,85,86] (Figure 2). This approach also enables the study of epigenetic contributions to amyloid progression by introducing (knock-in) mutations into distinct genetic and epigenetic backgrounds.

Genome editing tools—including zinc finger nucleases (ZFNs), transcription activator-like effector nucleases (TALENs), and most prominently, Clustered Regularly Interspaced Short Palindromic Repeats (CRISPR)-Cas9—have revolutionized stem cell research. In particular, their application in iPSC allows for the precise introduction or correction of disease-causing mutations within an otherwise isogenic genetic background, thereby eliminating confounding effects arising from inter-individual genetic variability. CRISPR-Cas9-based platforms, including nuclease-mediated editing, base editors, prime editors, and catalytically inactive Cas9 (dCas9) fusions for epigenetic modulation, provide versatile tools for genome manipulation [87]. Notably, CRISPR-Cas9 outperforms earlier technologies like TALENs in generating efficient non-homologous end joining (NHEJ)-mediated mutations, particularly at non-expressed loci in iPSCs, while achieving comparable efficiencies in homology-directed repair (HDR)-based editing [88]. Moreover, the guide RNA component of it can discriminate between alleles differing by a single nucleotide, enabling precise, allele-specific targeting of disease-associated mutations. This allows the generation of isogenic controls by correcting pathogenic alleles in patient-derived iPSCs (to test necessity) or introducing specific mutations into healthy iPSCs (to test sufficiency). Furthermore, this approach enables the introduction of knock-in or knock-out alleles for genes hypothesized to act as antagonistic or synergistic modifiers during disease progression, facilitating investigation into gene-gene and gene-environment interactions in a controlled isogenic context.

In liver-focused disease modeling, edited iPSCs will be differentiated into HLCs, providing a functional model for studying liver-specific disorders and hepatically secreted proteins, including TTR [89,90]. The CRISPR-iPSC platform has enabled modeling of several monogenic liver diseases, e.g., familial hypercholesterolemia [91] and alpha-1 antitrypsin deficiency [92]. In the case of ATTR, hepatocytes are the primary source of circulating TTR, making iPSC-HLCs an ideal cellular model for this condition. When CRISPR-Cas9 is applied at the iPSC stage, the resulting HLCs retain the intended genomic edit, enabling side-by-side comparisons of mutant and corrected cell lines. A compelling example of this approach is the study by Giadone et al. [77], which targeted the aggressive Leu55Pro mutation in TTR. Using HDR-mediated CRISPR-Cas9 editing, the Leu55Pro mutation was corrected in patient-derived iPSCs. Following differentiation into HLCs, mutant lines secreted aggregation-prone TTR oligomers that activated hepatic proteostasis responses, while corrected lines secreted mostly stable tetrameric TTR. The study also demonstrated that activation of the activating transcription factor 6 (ATF6) branch of the unfolded protein response could preferentially suppress the secretion of amyloidogenic TTR without affecting the secretion of wild-type TTR [77]. The platform also permits interrogation of mutation interactions; for instance, compound heterozygosity for Val30Met and Val122Ile mutations has been associated with more severe disease phenotypes and accelerated progression [93]. Using CRISPR-based editing, these interactions can be modeled by introducing multiple mutation combinations into an isogenic background, allowing mechanistic dissection of combinatorial effects. Together, these findings establish CRISPR-edited iPSC-HLCs as a powerful platform for modeling ATTR amyloidosis, offering genetically matched systems to dissect mutation-specific pathogenesis, gene-gene interactions, and responses to targeted interventions.

## 4. iPSC-Derived 3D Cardiac Models for ACM

**Figure 3:**
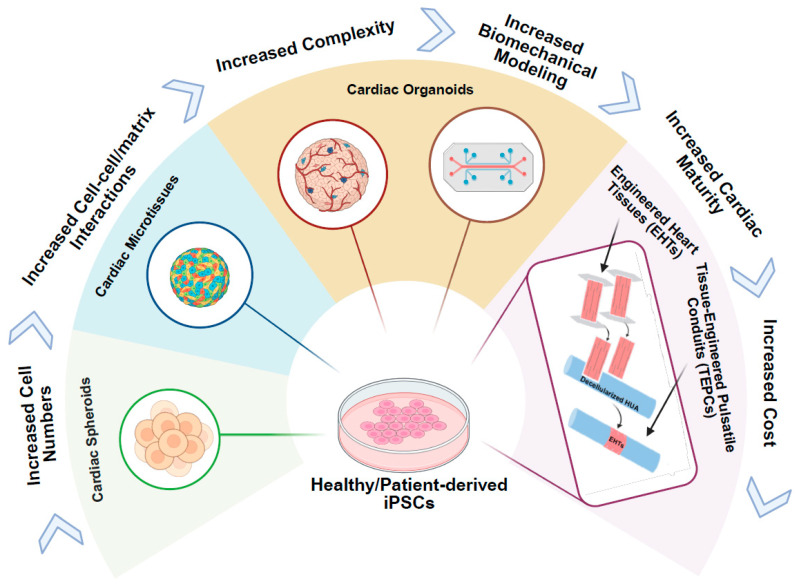
**iPSC-derived 3D cardiac tissue models for amyloid cardiomyopathy.** iPSCs are differentiated into key cardiac lineages—cardiomyocytes (CMs), cardiac fibroblasts (CFs), and endothelial cells (ECs)—and assembled into three-dimensional constructs, ranging from simple to complex tissue architectures that mimic native heart tissue. These engineered tissues are exposed to patient-derived amyloid proteins, either isolated directly from serum or urine (light chain or transthyretin) or delivered via conditioned medium from patient-specific iPSC-derived-HLCs secreting pathogenic amyloid protein. The 3D tissue model cardiac complexities, as well as cellular and mechanical abnormalities characteristic of amyloid cardiomyopathy, enabling the study of disease mechanisms in a physiologically relevant context. The direction of the arrows indicates a progression from simple to complex tissue architecture.

iPSCs can give rise to all major cardiac cell types—including cardiomyocytes, endothelial cells (ECs), and cardiac fibroblasts (CFs)—which are directly affected by amyloid infiltration in ACM [83]. This makes iPSC-derived cardiac cells particularly valuable for building multicellular, 3D tissue models, including spheroids, organoids, cardiac microtissues (cMTs), and engineered heart tissues (EHTs) that recapitulate the structural, mechanical, and electrophysiological environment of the human myocardium, while maintaining the patient’s genetic context (Figure 3). In contrast to traditional 2D monolayer cultures, 3D constructs—whether spheroids, organoids, cMTs, or EHTs—enable cell–cell and cell–matrix interactions, establish physiological diffusion gradients, and promote more mature cardiac phenotypes. This enhanced fidelity allows researchers to capture complex amyloid-driven processes, including extracellular fibril deposition, contractile dysfunction, calcium handling abnormalities, and electrophysiological remodeling, in a controlled and human-specific system. Studies have also revealed cell–type–specific phenotypes in ATTR cardiomyopathy: cardiomyocytes display sarcomere disarray, impaired calcium handling, and electromechanical uncoupling; endothelial cells exhibit reduced migratory capacity and abnormal morphology; while fibroblasts remain viable but show enhanced association with TTR fibrils [83]. These findings highlight the importance of 3D models that integrate multiple cardiac cell types to more accurately recapitulate the complex multicellular environment of the diseased myocardium Table 1.

### 4.1. Cardiac Spheroids

Cardiac spheroids derived from iPSCs have emerged as a promising 3D model to study ACM, offering structural and functional advantages over traditional 2D systems. These spheroids, composed primarily of iPSC-CMs and optionally incorporating CFs and ECs, better replicate the multicellular and biomechanical environment of native myocardium, where amyloid fibrils accumulate [94]. Notably, when compared to 2D monolayers, the 3D context of these spheroids enhances disease modeling fidelity by supporting cell–cell and cell-matrix interactions [110]. Further strengthening the physiological relevance of iPSC-CM spheroids, recent work has shown that cyclic uniaxial stretch and electrical stimulation significantly promote cardiomyocyte maturation. Stimulated spheroids exhibited increased expression of maturation markers (e.g., cTnI, MLC2v), improved sarcomere organization, and enhanced electrophysiological performance, including the ability to follow pacing up to 4 Hz [95].

In a recent study modeling AL amyloidosis, Bézard et al. generated iPSC-CM spheroids co-cultured with primary human cardiac fibroblasts and then exposed them to patient-derived amyloidogenic immunoglobulin light chains, generating cardiac spheroids within 7 days. These spheroids uniformly distributed LCs throughout the tissue and displayed hallmark pathological features such as disorganized sarcomeric α-actinin, SERCA2a accumulation, impaired contractility, and altered calcium transients across multiple patient-derived LC variants [94]. Similarly, multicellular iPSC-CM spheroids exposed to recombinant TTR fibrils (wild-type, Val122Ile, or Val30Met) showed decreased cell viability, sarcomere disorganization, prolonged calcium transients, and disrupted electromechanical coupling—mirroring early pathophysiological changes observed in ATTR [83]. Together, these studies support the use of cardiac spheroids as a physiologically relevant 3D model for both AL and ATTR, thus enabling sensitive and spatially resolved assessment of amyloid-induced cardiotoxicity. This includes contractile dysfunction, calcium dysregulation, and structural degeneration, within a multicellular human cardiac environment that more closely mimics in vivo tissue architecture.

### 4.2. Cardiac Microtissues

cMTs derived from iPSCs provide a scalable and physiologically relevant 3D platform for modeling ACM [96]. These models typically integrate cardiomyocytes with stromal and endothelial components, enabling the structural organization, electromechanical coupling, and cell–cell interactions that better mimic the in vivo myocardium [97,98,99] (Figure 3, Table 1).

Recent applications of cMTs have demonstrated their utility in modeling inflammation-driven cardiac injury, chamber-specific electrophysiology, and vascular remodeling—features directly or indirectly relevant to cardiac amyloidosis. For instance, disease-specific cMTs exposed to pro-inflammatory signals recapitulate pathological hallmarks such as capillary proliferation, metabolic dysfunction, and contractile impairment [99]. Similarly, chamber-specific atrial cMTs exhibit distinct action potential properties and drug responses [98], highlighting the potential of cMTs to model atrial-selective vulnerability in amyloid disease. These platforms also support high-content readouts, including calcium transients, sarcomere structure, and electrophysiological profiling, facilitating mechanistic studies and therapeutic testing. Together, these findings position cMTs as a flexible and informative model for investigating ACM across genetic, inflammatory, and electrophysiological dimensions.

### 4.3. Cardiac Organoids

hiPSC-derived cardiac organoids are emerging as versatile 3D models for studying ACM due to their ability to recapitulate the structural and functional complexity of native cardiac tissue. These organoids, generated through self-organization or engineered assembly, typically consist of cardiomyocytes, CFs, ECs, and occasionally specialized populations such as pacemaker-like or neuronal cells [101,102]. They demonstrate autonomous beating, chamber-like morphogenesis, and vascular network formation, reflecting key aspects of human cardiac development and physiology (Figure 3, Table 1). Optimization of Wnt signaling, geometric constraints, and co-culture strategies has been shown to enhance organoid maturation, yielding organized sarcomeres, synchronized contractions, and electrophysiological responses comparable to in vivo myocardium [101]. In more recent models, tissue-resident macrophages from hemogenic endothelium within cardiac organoids have been demonstrated, enabling the study of immune-cardiac interactions [111].

Organoid model also enables the study of multi-organ interaction through either self-assembly (multilineage organoids, assembloids) or microfluidic device (organ-on-a-chip) (Figure 3) [103]. While cardiac organoids have been previously used in liver–heart co-culture systems to assess metabolism-dependent drug toxicity [104], a recent application of this technology to ATTR cardiomyopathy demonstrates the power of organoid platforms. Using a microfluidic co-culture system, hepatic organoids expressing the pathogenic TTR-Val122Ile variant were connected to cardiac organoids to simulate physiological circulation. The hepatocytes secreted TTR-Val122Ile at concentrations seen in patients (3–7 µM), which was taken up by cardiomyocytes, inducing hallmark ATTR features such as oxidative stress, cytotoxic protein aggregation, impaired diastolic relaxation, and electrical conduction abnormalities. Functional assessments confirmed diastolic dysfunction and abnormal calcium handling in the cardiac organoids, closely mirroring clinical observations in ATTR patients [105]. This heart–liver organoid system represents a powerful new tool for modeling hereditary ATTR in vitro, enabling the visualization of organ interactions.

### 4.4. Engineered Heart Tissues

Among 3D cardiac models, EHTs offer a uniquely powerful platform for modeling amyloid cardiomyopathy due to their capacity to mimic not only cellular and tissue architecture but also the biomechanical properties of the myocardium. Unlike simple cardiac microtissues, EHTs incorporate mechanically constrained geometries and tunable extracellular matrices, enabling alignment, force generation, and mechanical load—all of which are crucial for modeling the skewed contractile function observed in amyloid-infiltrated myocardium (Figure 3, Table 1). A compelling example of EHT utility comes from a study by Riaz et al., where patient-derived iPSC-CMs with myosin heavy chain 7 (MYH7) and muscle LIM protein (MLP) mutations were seeded into laser-cut scaffolds mimicking native cardiac fiber alignment. Under mechanical loading, these EHTs displayed impaired contractile force generation, sarcomere disorganization, altered calcium handling, and dysregulation of mechanotransduction-related genes. Mechanistic analysis revealed that excessive crossbridge formation and slowed relaxation destabilized the Z-disc MLP complex, triggering calcineurin–NFAT signaling and hypertrophy [78].

This work illustrates how patient-derived EHTs can recapitulate complex genotype–phenotype relationships in a mechanically dynamic human cardiac context—an approach directly applicable to amyloid cardiomyopathy, where amyloid similarly disrupts sarcomeric integrity and force sensing. For example, tissues engineered from hiPSC-CMs exhibit periodic myofilament lattice organization [112], with contractile force inversely correlated with lattice spacing and directly with cross-bridge density—parameters that are expected to deteriorate with amyloid infiltration. Moreover, EHTs allow integration of patient-specific mutations and extracellular stimuli to mimic both hereditary (e.g., TTR) and acquired (e.g., AL) disease forms. This makes EHTs an ideal platform not only for dissecting pathogenic mechanisms of amyloid aggregation at the nanoscale but also for screening candidate therapeutics aimed at restoring contractile balance and sarcomere architecture. Beyond this, EHTs can be subjected to controlled electrical and mechanical conditioning that mimics physiological stimuli, further promoting cardiac maturation [107,113]. Extending this concept, Tissue-Engineered Pulsatile Conduit (TEPC) systems—constructed by wrapping EHTs around decellularized human umbilical arteries (HUAs)—not only preserve stretch and electrical conduction within the EHT compartment but also generate luminal pressure, effectively reproducing the pumping function of the heart [79,108] (Figure 3).

A recent advance in this area is the development of a fully human 3D ATTR model that incorporates the principal hepatic and cardiac cellular players alongside a representative extracellular environment. In this approach, an iPSC line derived from a patient carrying the TTR-Ser43Asn mutation was differentiated into hepatocytes, cardiomyocytes, and CFs, each embedded in tailored hydrogels and integrated into a melt electro-writing (MEW)–fabricated scaffold. These multi-lineage tissues maintained functional outputs—albumin secretion from hepatic constructs and spontaneous contraction from cardiac constructs—over four weeks in culture, while displaying spatially uniform cell distribution and mature lineage markers. Upon exposure to TTR fibrils, cardiac tissues exhibited shortened calcium transients, consistent with altered excitation–contraction coupling. However, beat rate, metabolic activity, and gene expression remained unchanged at the tested concentrations [114]. By integrating hepatic TTR production with cardiac functional readouts in a single engineered platform, this model recapitulates key aspects of ATTR pathophysiology and provides a foundation for mechanistic studies and therapeutic discovery in a human-relevant context.

## 5. iPSC-Derived Models and Mechanistic Studies of AL and ATTR

The experimental modeling of ACM has progressed from simple invertebrate systems capturing core proteotoxic mechanisms to complex mammalian and human-derived platforms capable of reproducing tissue-specific cardiac pathology. hiPSC-CM models, in both 2D culture and 3D tissue, have transformed ACM research by enabling human-specific, mechanistic studies that bridge molecular pathology to functional cardiac impairment.

### 5.1. Evolution of ACM Models

Early efforts to model ACM relied on invertebrate and vertebrate systems, each contributing unique insights but also illustrating key limitations when translated to human disease. Invertebrate models—notably *Drosophila* expressing human TTR-V30M—revealed age-dependent motor deficits, shortened lifespan, and Congo-red-positive aggregates, highlighting cell-autonomous toxicity of misfolded TTR [115]. Similarly, a *C. elegans* model driving TTR expression in muscle recapitulated secretion defects and nociceptive neuron pathology, demonstrating cell-nonautonomous amyloid toxicity [116,117]. Zebrafish offer a rapid vertebrate system for AL cardiotoxicity: injection of patient-derived amyloidogenic light chains into 48 hpf larvae causes pericardial edema, impaired cardiac function, and mortality within two weeks [118].

Rodent models began with transgenic mice overexpressing TTR-Val30Met, which yielded only sparse amyloid in the kidney, gut and cardiovascular organs, without peripheral neuropathy involvement [119]. Overexpression often required crossing onto mouse TTR-knockout backgrounds or deletion of stress-response regulators (e.g., HSF1) to achieve fibrillogenesis, revealing how hepatic unfolded protein response (UPR) and chaperone dynamics modulate deposition [120,121]. More recently, humanized “knock-in” strains at the TTR and retinol-binding protein 4 (RBP4) loci have developed age-dependent amyloid in the gut, heart, and even sciatic nerve perineurium, underscoring the importance of species-matched partners in TTR aggregation [122]. Similarly, for AL-ACM, a novel transgenic mouse model expressing a patient-derived human λ light chain (λS-LC) demonstrated that while full-length LC alone was not overtly toxic, the administration of preformed LC fibrils or unstable LC fragments triggered systemic amyloid deposition and early cardiac dysfunction [123]. This model mirrors key features of human AL amyloidosis and highlights LC proteolysis as a critical initiator of fibrillogenesis, offering a robust tool for mechanistic and therapeutic investigations.

Non-human primates, such as aged vervet monkeys carrying the human TTR-Val122Ile allele, spontaneously develop cardiac arrhythmias and fibril deposits, providing a physiologically faithful—but logistically challenging—model for wild-type amyloidosis [124]. These animal systems laid crucial groundwork but fell short in capturing human cardiomyocyte–specific responses and organ-level mechanics. For a more detailed review of the animal models established for investigating ACM disease progression, the reader is referred to [125].

### 5.2. Biomarkers in AL and ATTR

Cardiac biomarkers such as high-sensitivity cardiac troponin T (hs-cTnT) and N-terminal pro-brain natriuretic peptide (NT-proBNP) are reliable indicators of myocardial involvement and prognosis in both AL and ATTR cardiomyopathy [126,127,128,129]. Accordingly, the Mayo Clinic first proposed a three-stage system in 2004 based on troponin T (TnT < 0.035 µg/L) and NT-proBNP (<332 ng/L): stage I if both markers were below the thresholds, stage II if only one was elevated, and stage III if both were elevated [130]. The immunoglobulin free light chain (FLC) is specific as a critical prognostic marker in AL patients, with higher baseline FLC levels correlating to increased mortality risk, greater organ involvement, and advanced disease stage. Post-treatment normalization of FLC is a stronger predictor of survival and organ response than hematologic response alone [131]. Therefore, the Mayo Clinic staging model was revised in 2012 to include the difference in free light chains (dFLC ≥ 180 mg/L) as an additional marker, assigning one point each for TnT ≥ 0.025 ng/mL, NT-proBNP ≥ 1800 pg/mL, and dFLC ≥ 180 mg/L, stratifying patients into stages I–IV [128]. In 2015, a European group modified the 2004 Mayo system to better identify very-high-risk patients, subdividing stage III into IIIa and IIIb according to an NT-proBNP threshold of 8500 pg/mL [132].

In ATTR amyloidosis, biomarkers like RBP4, serum TTR levels, and novel staging tools involving estimated glomerular filtration rate (eGFR) and NT-proBNP also help predict outcomes [133,134,135]. Genetic testing, including next-generation sequencing panels of amyloidogenic genes, aids in diagnosing hereditary amyloidosis [136]. Emerging biomarkers related to TTR kinetic stability and non-native TTR species show promise for early detection and monitoring of treatment response in ATTR.

### 5.3. Current Mechanistic Studies in AL and ATTR

Emerging evidence challenges the earlier assumption that cardiac amyloidosis results solely from passive amyloid deposition. Instead, studies show that soluble amyloidogenic LCs and misfolded TTR proteins can exert direct proteotoxic effects on cardiomyocytes, independent of fibril formation. In AL amyloidosis, amyloidogenic LCs isolated from patients with cardiac involvement increase intracellular reactive oxygen species (ROS) and induce the expression of heme oxygenase-1, a redox-sensitive stress protein, in rat isolated cardiomyocytes. This oxidative stress leads to impaired calcium handling, disrupted contractility, and cardiomyocyte apoptosis [19]. These effects are mediated through a noncanonical activation of the p38α Mitogen-Activated Protein Kinase (MAPK) pathway, where transforming growth factor β-activated kinase 1-binding protein 1 (TAB1) drives p38α autophosphorylation, independent of Mitogen-Activated Protein Kinase Kinase 3 and 6 (MKK3/6). Inhibition of this pathway with SB203580, a selective p38 MAPK inhibitor, reduces oxidative stress and improves cardiomyocyte survival [20]. Further mechanistic work shows that LCs are internalized by cardiomyocytes and localize to mitochondria and lysosomes, leading to mitochondrial dysfunction, decreased ATP production, and impaired autophagy. Dysfunctional lysosomes result in accumulation of misfolded proteins, perpetuating a feedforward loop of proteotoxic stress [137]. Similar downstream effects occur in ATTR. Although mutant TTR toxicity appears more extracellular [138], it also culminates in mitochondrial injury, oxidative damage, and impaired sarcomere integrity [139], suggesting a shared final common pathway of mitochondrial-driven cardiotoxicity in both AL and ATTR.

### 5.4. Role of iPSC-CMs as 2D and 3D Models for Mechanistic Studies of ACM

Patient-specific iPSC models addressed many of these gaps, providing human cardiomyocyte–specific responses and organ-level mechanics. In 2D monolayer cultures, cardiomyocytes exposed to patient-derived recombinant amyloidogenic TTR variants (e.g., Val122Ile, Val30Met) reproduce hallmark features of proteotoxicity—sarcomeric disorganization, calcium dysregulation, oxidative stress, and apoptosis—in a genetically controlled, human-specific context [69,70]. These systems enable high-throughput screening of pathway inhibitors and functional assays such as calcium transient and metabolic analysis.

3D models—including spheroids, organoids, cMTs and EHTs—further enhance physiological relevance by incorporating multicellular composition, cell–matrix interactions, physiological diffusion gradients, and mechanical loading, all of which modulate stress pathway activation. Among these, EHTs uniquely combine multicellular composition with defined geometry, mechanical preconditioning, and direct force measurement, allowing mechanistic dissection of how amyloid fibrils compromise sarcomeric integrity, reduce contractile force, and stiffen tissue [140,141].

Advanced characterization pipelines further expand the mechanistic depth of these models. Electrophysiological mapping of EHTs with voltage-sensitive dyes enables the precise quantification of conduction velocity, resting membrane potential, and action potential duration, linking electrical deficits to structural changes such as reduced connexin-43 coupling [114,142]. Biomechanical profiling captures twitch force, time to peak, and relaxation time, while ratiometric calcium imaging provides simultaneous measurements of calcium transient amplitude and kinetics [78]. Mitochondrial metabolism [143] can be interrogated using Seahorse-based stress testing to quantify basal respiration, ATP-linked respiration, maximal respiratory capacity, and spare capacity, allowing for the detection of amyloid-induced metabolic collapse or recovery following interventions.

At the molecular level, single-cell RNA sequencing (scRNA-seq) of cardiac constructs [144,145] can resolve cell-type–specific transcriptional responses to amyloid stress, identify vulnerable subpopulations, and uncover dysregulated pathways in excitation–contraction coupling, mitochondrial function, and proteostasis. Bioinformatic integration with patient datasets enables mapping of in vitro phenotypes to clinical signatures. Key transcriptional and functional findings can be validated through bulk RNA analysis, pharmacological inhibition, or gene knockdown approaches to confirm pathway causality.

Together, iPSC-based 2D and 3D models—complemented by integrated electrophysiological, biomechanical, metabolic, and transcriptomic profiling—offer a multi-layered, human-specific framework for elucidating how amyloidogenic proteins drive oxidative stress, mitochondrial dysfunction, calcium overload, and maladaptive signaling, ultimately culminating in the contractile and conduction abnormalities that define AL and ATTR.

## 6. iPSC-Derived Platforms for Drug Screening to Prevent ACM

Current therapeutic approaches for ATTR can be categorized into three main groups, each targeting a distinct stage of the amyloidogenic cascade. Protein stabilizers—including tafamidis, diflunisal, and acoramidis—bind to the thyroxine-binding sites of circulating TTR tetramers, preventing their dissociation into monomers and subsequent misfolding into amyloid fibrils. Tafamidis, the first approved therapy for ATTR, demonstrated substantial reductions in all-cause mortality and cardiovascular hospitalizations in the clinical trial, with benefits weakened by late initiation [37]. Acoramidis, a newer stabilizer with enhanced binding potency, has also shown favorable effects on clinical outcomes, particularly reducing hospitalizations [146]. Gene-silencing strategies aim to lower circulating TTR levels by inhibiting hepatic synthesis, thereby limiting substrate availability for fibril formation. These include small interfering RNAs (siRNAs) such as patisiran [147] and vutrisiran [148] and antisense oligonucleotides (ASOs) such as inotersen and eplontersen [149], with clinical trials demonstrating improvements in functional capacity, biomarkers, and, in some cases, imaging parameters. In addition, CRISPR–Cas9-based gene-editing therapies, such as nexiguran ziclumeran [150], are in advanced clinical development and hold promise as single-dose, long-lasting treatments. Finally, amyloid clearance agents target existing fibril deposits to promote organ recovery. Monoclonal antibodies like ALX2220 and coramitug, as well as fusion constructs like AT-02, engage immune-mediated phagocytosis to remove amyloid, with early-phase trials reporting encouraging biomarker and imaging improvements [14].

While these therapeutic strategies represent significant progress, their optimal deployment—and the development of next-generation interventions—requires robust, human-relevant preclinical models capable of capturing the complex hepatic–cardiac interplay and patient-specific disease mechanisms in amyloid cardiomyopathy. iPSC-derived HLCs and cardiomyocytes, used in both 2D and advanced 3D configurations, provide a precisely such a platform, enabling the mechanistic dissection and systematic screening of candidate compounds across the entire amyloidogenic cascade. In 2D systems, iPSC-HLCs enable the upstream screening of gene-silencing or gene-editing approaches and small molecules that reduce amyloidogenic TTR production [89,90]. Meanwhile, iPSC-CMs enable the direct evaluation of cardiotoxicity mitigation, rescue of contractile function, and reversal of calcium handling defects [140]. Furthermore, transitioning to 3D formats significantly improves physiological relevance. For example, EHTs derived from patient-specific iPSC-CMs can replicate sarcomere disorganization, contractile impairment, and electrophysiological remodeling observed in amyloid-infiltrated myocardium. Additionally, EHT array platforms offer semi–high-throughput, quantitative readouts for assessing multiple candidate compounds parallel [141]. Similarly, advanced hepatic constructs—either stand-alone or integrated into multi-organ “liver–heart-on-chip” systems—sustain long-term, physiologically relevant TTR or LC secretion, enabling direct correlation of upstream therapeutic suppression with downstream preservation of cardiac function [50]. By integrating 2D and 3D cardiac and hepatic models into unified pipelines, these iPSC-based platforms offer a human-relevant, scalable, and mutation-specific approach for pharmacogenomic stratification and high-content drug screening, paving the way for more precise and effective treatments for ACM.

## 7. Limitations of iPSC-Based Models for ACM Research and Future Perspectives

Despite their transformative potential, iPSC-based models for ACM face several intrinsic limitations that must be acknowledged when interpreting results or designing experiments. A major challenge lies in the developmental immaturity of iPSC-CMs. These cells, even after extended culture duration or within 3D constructs, retain a fetal- or neonatal-like phenotype in multiple dimensions. Structurally, they exhibit smaller, rounder morphology with disorganized sarcomeres and underdeveloped transverse-tubule (T-tubule) networks. Electrophysiologically, they display depolarized resting membrane potentials, slower upstroke velocities, and prolonged action potential durations compared to adult ventricular cardiomyocytes. Metabolically, they rely predominantly on glycolysis rather than the oxidative phosphorylation-driven, fatty acid-based metabolism characteristic of mature myocardium. Functionally, their contractile force and calcium handling kinetics are reduced, and their responses to β-adrenergic stimulation are blunted [151].

In contrast, ACM—whether ATTR or AL—is primarily a late-onset disease, where cardiomyocytes have undergone decades of structural remodeling, cumulative oxidative stress, and progressive decline in proteostatic capacity [1,15,33]. This temporal disconnect raises questions about how accurately immature iPSC-CMs and derived 3D models can recapitulate age-dependent pathogenic mechanisms, including altered protein homeostasis and mitochondrial vulnerability seen in elderly hearts.

To bridge this maturity gap, multiple strategies have been developed. More efficient and robust cardiac differentiation protocols have now been developed to generate chamber-specific, mature cardiomyocytes [112,113,152], which can be further developed into 3D models for more accurate ACM modeling, mechanistic insights, and the discovery of effective drugs. Prolonged culture durations (up to several months) promote progressive sarcomeric organization and metabolic shifts, further enhanced via electrical pacing [153]. Metabolic conditioning, such as fatty acid [154] or retinoic acid [152] supplementation, promotes oxidative metabolism in iPSC-CMs. Hormonal treatments—particularly triiodothyronine (T3) and glucocorticoids—enhance T-tubule formation and excitation–contraction coupling [155]. Tissue-engineered models, especially EHTs, combine aligned architecture, preload/afterload conditions, and chronic electromechanical stimulation to achieve superior structural and functional maturation. Electrical stimulation at physiological pacing rates improves excitation–contraction coupling, while cyclic mechanical stretch enhances myofibrillar alignment and force generation [79,108,109,156].

Still, these approaches do not fully reproduce the “aged” cardiac phenotype relevant to ACM, especially in the ATTR-wt context. In vitro aging strategies are emerging, including long-term culture with oxidative stressors [157], telomere shortening via telomerase reverse transcriptase suppression [158], and targeted mitochondrial DNA damage to model age-related bioenergetic decline [159]. Such interventions aim to introduce the chronic stress landscape that aged myocardium endures, potentially unmasking late-onset amyloid susceptibility.

Additional challenges stem from the fact that reprogramming resets epigenetic aging marks, erasing donor age–related transcriptional and proteostatic signatures [160]. Furthermore, most current systems lack the multicellular complexity of native myocardium—omitting CFs, ECs, immune cells, and conduction system elements that may modulate amyloid deposition, inflammation, or electrophysiological vulnerability. Equally important, the systemic nature of ACM is challenging to model in isolated cardiac cultures: in ATTR, the hepatic secretion of mutant TTR drives the cardiac amyloid burden, while in AL, clonal plasma cell populations produce cardiotoxic light chains. Multi-organ-on-a-chip platforms and microfluidic co-cultures of iPSC-derived HLCs, cardiomyocytes, and immune components are beginning to address this gap [161,162].

Looking to the future, the integration of patient-specific iPSCs with CRISPR-based isogenic editing, vascularized cardiac organoids, immune-competent co-cultures, and multi-organ micro-physiological systems is poised to redefine how we model ACM. Incorporating features of biological aging and maturation—through biomechanical stimulation, electrical pacing, and metabolic conditioning—may be pivotal in capturing the complex, age-related proteotoxic mechanisms that drive disease onset and progression. These advances will not only deepen mechanistic understanding but also catalyze the development of predictive, human-relevant platforms for high-throughput drug screening and precision medicine. Ultimately, such systems have the potential to accelerate the translation of targeted therapies from the bench to the bedside, thereby transforming care for patients with AL and ATTR cardiomyopathy.

## Figures and Tables

**Table 1:** Comparison of iPSC-Derived 3D Models for ACM.

Model	Cardiac Spheroids [94,95]	Cardiac Microtissues (cMTs) [96,97,98,99,100]	Cardiac Organoids [101,102,103,104,105,106]	Engineered Heart Tissues (EHTs) [78,79,107,108,109]
Similarities	1. Multicellular Integration—All models can incorporate multiple cell types (CMs, CFs, ECs) to mimic the cellular diversity of myocardium; 2. Cell–Cell and Cell–Matrix Interactions—Each model reproduces key intercellular and matrix interactions, essential for structural and functional fidelity; 3. Physiological Gradients—All platforms establish oxygen, nutrient, and signaling diffusion gradients absent in 2D cultures, thereby promoting more in vivo–like physiology; 4. Enhanced Maturation—Compared to 2D iPSC-CMs, all 3D models promote structural and functional maturation, including improved sarcomere alignment, calcium handling, and metabolic activity; 5. Disease Modeling Fidelity—all allow mechanistic dissection of amyloid-induced toxicity within a physiologically relevant 3D human cardiac context.			
Differences	Simplest structure with less cells; compact aggregates; lack spatial organization and chamber-like structures seen in organoids.	Engineered, reproducible constructs with controlled geometry; smaller and simpler than organoids.	Self-organizing, higher complexity; can form chamber-like structures and vascular networks; enable microenvironmental control via microfluidic systems.	Structured tissues constrained by scaffolds; allow mechanical preconditioning and direct force measurement.
Special Advantages	Easy to generate, reproducible, allow contractility, calcium handling, and sarcomere disorganization studies under amyloid stress.	Scalable and compatible with high-content assays; support chamber-specific modeling (atrial vs. ventricular).	Enable multi-lineage and multi-organ modeling (e.g., liver–heart co-culture for TTR secretion and cardiotoxicity).	Provide biomechanical readouts (force, TTP, RT50); allow electromechanical training; mimic amyloid-induced contractile dysfunction.
Subtypes/Examples	Mono-/multicellular spheroids; stretched/electrically stimulated spheroids.	Chamber-specific atrial/ventricular microtissues.	Cardiac-only organoids; multi-lineage organoids; assembloids; organ-on-a-chip systems.	Biowire platform; mechanically/electrically conditioned EHTs; Tissue-engineered pulsatile conduits (TEPCs).

## Data Availability

No new data were created or analyzed in this study. Data sharing is not applicable to this article.
